# Overexpression of RAPGEF3 enhances the therapeutic effect of dezocine in treatment of neuropathic pain

**DOI:** 10.1590/1678-4685-GMB-2020-0463

**Published:** 2021-11-19

**Authors:** Xue Liu, Li Song, Xiaojun Ma, Yong Liu, Hui Huang, Yongsheng Xu, Wei Yan

**Affiliations:** 1 The Affiliated Hospital of Qingdao University, Department of Anesthesiology, Qingdao, Shandong, China.

**Keywords:** RAPGEF3, dezocine, neuropathic pain, Ras/p-38 MAPK, inflammation

## Abstract

Pain is a significant problem worldwide that affects the quality of life of patients. Dezocine is a non-addictive analgesic drug with kappa-opioid antagonist activity and has been successfully used to alleviate of postoperative pain. In addition, dezocine has an analgesic effect similar to that of morphine, alleviating moderate to severe pain. Rap guanine nucleotide exchange factor 3 (RAPGEF3) is a guanine nucleotide exchange factor for GTPases Rap1 and Rap2, which could enhance the activity of Rap1 to promote cell adhesion and axon regeneration, as well as promote neurite extension by interacting with nerve growth factors. Here, we first observed that overexpression of RAPGEF3 increased cell viability, as shown by a CCK-8 assay, and recovered brain function in rats. The expression of inflammation-related factors at the mRNA level was detected using qPCR, and the concentration of these factors in a cultured cell medium and rat serum samples were decreased as shown by ELISA after RAPGEF3 overexpression. Through western blotting, we further found that pro-inflammatory proteins were decreased, and these effects might be mediated by inhibition of the Ras/p-38 MAPK signaling pathway. Taken together, we speculated that RAPGEF3overexpression enhances the therapeutic effect of dezocine on neuropathic pain by inhibiting the inflammatory response through inhibition of the Ras/p-38 MAPK signaling pathway.

## Introduction

Neuropathic pain (NP) is normally caused by lesions in the nervous system and leads to long-term debilitating conditions, which reduce the quality of life. Pathophysiological changes in patients with NP result in anxiety, depression, and elevated blood pressure (Kroth *et al.*, 2020). There are several types of NP, including painful non-diabetic neuropathy, trigeminal neuralgia, multiple sclerosis, and spinal cord injury (Bouhassira, 2019). NP is a commonly encountered disease, but effective treatment for patients with NP still lacks due to the adverse effects of treatment drugs. Dezocine, also known as a partial μ opioid receptor agonist and κ receptor agonist, is a new narcotic analgesic that has been widely used to treat anesthesia analgesia, with clinical trials to treat neuropathic pain having been conducted (Zhang et al., 2019). Dezocine has been used to reduce postoperative pain after surgery at a regular dose of 5-10 mg (Pandit et al., 1986), and its effect in treating moderate to severe pain has been considered approximately equal to that of morphine, without a tolerance effect (Walker et al., 1999). A previous study found that the combination of dezocine and midazolam has a treatment effect in relieving pain and anxiety in patients (Walker *et al.*, 1999). Other studies showed that dezocine antagonizes morphine analgesia in acute nociception (Li et al., 2017), and could be successfully applied to anesthetize of indolent colonoscopy patients without pain in combination with propofol (Bos, 2006). Cyclic adenosine monophosphate (cAMP) is a well-known regulator of biological processes, including gene transcription, cell proliferation, migration and apoptosis. Rap guanine nucleotide exchange factor 3 (RAPGEF3) was first identified in 1998 as a new cAMP sensor of cAMP (Bramanti et al., 2015), and has been regarded as an important regulator of many cellular processes, such as survival, proliferation and neuronal signaling (Xu et al., 2016a). Using a RAPGEF3 deletion mouse model, researchers observed increased expression of RAPGEF3 in the root ganglia (DRG) after nerve transection, reducing the inflammatory mediator as well as NLRP3 inflammasome (Carswell et al., 1975). In addition, overexpression of RAPGEF3 has been found to promote the neointimal formation in aortic tissues (Chaplan et al., 1994). RAPGEF3 regulates inflammation and plays a critical role in inflammatory pain through the ERK signaling pathway, and has been regarded as a therapeutic target for chronic pain (Pan *et al.*, 2019). However, the function of RAPGEF3 in NP remains unclear. 

## Material and Methods

### Reagents

H-DMEM (11965092) and FBS (10093) were purchased from Thermo Fisher Scientific (Waltham, MA, USA). BsmBI (R0739S), KpnI (R3142S), and BamHI (R3136S) were purchased from Mew England Biolabs (Ipswich, MA, USA). Dezocine was acquired from the Dezocine Yangtze River Pharmaceutical Group (Jiangsu, China). TNF-α (H8916), IL-1β (SRP6169), CCL2 (MSST0036), and CXCL1 (SRP3216) were purchased from Sigma-Aldrich (St Louis, MO, USA). Anti-SOCS3 (ab16030), Epac1 (ab21236), GRK2 (ab137666), PDE4B (ab14611), PACAP (ab181205), PKCα (ab32376), p38 (ab31828), p38 (phospho T180 + Y182) (ab4822), Ras (ab180772), Raf (ab137435), RAP1(ab113480) antibody and IL-1β (ab197742), IL-6 (ab100713), TNF-α (ab208348) and COX-2 (ab210574) ELISA kits were purchased from Abcam (Cambridge, UK). 

### Cell culture

Primary glial cells were acquired previously described (Han et al., 2018). The cerebral cortex was obtained from newborn SD rats (2-4 days old, housed under 50-60% humidity, 22-24 ℃ atmosphere with a 12 h light/dark cycle, water and food freely available), and the cerebral cortex was removed and digested using 0.25% trypsin (supplied with 0.4% DNase). Digested glial cells and 293T cells were cultured in an H-DMEM medium with 10% FBS under a humidified 37 ℃ atmosphere supplied with 5% CO_2_. After 2 days of culture, the medium was changed to remove unattached cells.

### Vector construction

The full-length *RAPGEF3* gene was acquired using PCR with the following primers: Forward: 5′-CCGAAGCTGCTCCTACCA-3′, Reverse: 5′-ACTCCTCGCTGTTGGTGAGT-3′ (reaction steps: 50 ºC for 30 min and 85 ºC for 5 min. Amplified size: 594bp). Thereafter, the PCR products and pcDNA3.1 blank vector were digested with KpnI and BamHI, and linked with DNA ligase. Next, the pcDNA3.1-*RAPGEF3* overexpression vector was transfected into primary glial cells using Lipofectamine 2000 Transfection Reagent for 48 h, and the stably expressed cells were screened using G418. The *RAPGEF3* knockdown vector was constructed according to a previous study (Liu et al., 2017) with the following pair of primer: Forward: 5′-CAGAGAGGCGGCGATGCCAC-3′, Reverse: 5′-CAGAGAGGCGGCGATGCCAC-3′. Briefly, oligos were first annealed following the instructions, and blank vectors of lenti-CRISPR v2 and annealed oligos were digested with BsmBI for 30 min at 37 ℃. After linking with T4 PNK, the *RAPGEF3* knockdown vector was transfected into 293T cells to construct *RAPGEF3* knockdown lentivirus to transfect primary glial cells, and the stably expressed cells were screened using 2 μg/mL puro. 

### CCK-8 assay

The CCK-8 assay was performed according to the manufacturer’s instructions (CA1210; Solarbio Life Sciences, Beijing, China). Briefly, cells were seeded into each well of a 96-well plate at a concentration of 1×10^4^, and the cells were grouped and treated as previously described. Cells were then incubated with CCK-8 reagent for 4 h, and the OD value at 450 nm was detected using a SpectraMax iD3 microplate reader (Molecular Devices, San Jose, CA, USA). 

### Model construction

After the confluence of glial cells reached 70-80%, cells were treated with TNF-α (10 ng/mL), IL-1β (10 ng/mL), CCL2 (100 ng/mL), and CXCL1 (312 ng/mL) to mimic the inflammatory conditions of neuropathic pain before performing subsquent experiments. The cells were then divided into four groups: normal group (NC), dezocine treatment group (DT), dezocine treatment combined with RAPGEF3 overexpression group (DO), and dezocine treatment combined with RAPGEF3 inhibition group (DI). In the dezocine treated group, the cells were treated with 9 ng/mL dezocine for 10 consecutive days. Ten SD rats, five RAPGEF3 overexpression and five RAPGEF3 knockdown rats were purchased from Cyagen (Santa Clara, CA, USA). Rats were kept in a 22-24 ℃, 50-60% humidity atmosphere with a 12-h light/dark cycle, with freely available water and food. The neuropathic pain after sciatic nerve injury model was constructed according to a previous study (Ding et al., 2017). Briefly, rats were first anesthetized with isoflurane, and then the left sciatic nerve was loosely ligated using 4-0 chromic gut sutures at four segments 1 mm apart. Next, the sutures were tightened gently until a brisk twitch of the left hind limb was observed. The same procedure was performed on the rats in the sham group except for sciatic nerve ligation. Rats were divided into four groups: sham group (SH), dezocine treatment group (TD), dezocine treatment combined with *RAPGEF3* overexpression group (TO), and dezocine treatment combined with *RAPGEF3* inhibition group (TI). In the dezocine treated group, rats were injected with dezocine via intraperitoneal injection at a concentration of 3 mg/kg 30 min before the procedure.

### Behavioral testing

Mechanical allodynia in rats was detected using von Frey filaments. Rats in each group were divided and treated as previously described, and then the rats were placed in transparent Plexiglas compartments on a metal mesh. The surface of the left paw was subjected to von Frey hairs with logarithmically increasing stiffness (0.4-26 g). Rapid withdrawal or flinching of the paw was considered a positive response. Thereafter, the 50% paw withdrawal threshold (PWT) was detected using Dixon’s up-down method (Yokoyama et al., 2008). Thermal hyperalgesia was detected using a Hargreaves apparatus with radiant heat and presented as paw-withdrawal latency (PWL). Briefly, rats were placed in hyaline plastic compartments, while the left hind paw was exposed to a source of radiant heat via a glass plate within 20 s. 

### Ethical statement

The experiments were performed in accordance with the guidelines of the ethics committee of the Affiliated Hospital of Qingdao University (Qingdao, Shandong, No.SCNU-SLS-2017-03), and all animals were treated under the guidelines of the Public Health Service Policy on the Humane Care and Use of Laboratory Animals of the National Institutes of Health.

### RNA extraction

Rats and glial cells were grouped and treated as previously described, and the RNA extraction process was performed according to the protocol of the Total RNA Extraction Kit (R1200, Solarbio Life Sciences). Briefly, the cells and brain tissues of rats were lysed with lysis buffer, followed by incubation at room temperature for 5 min. After incubation with chloroform, the samples were centrifuged at 12,000 rpm for 5 min (4 ℃). The water phase of the samples was then removed into the absorption tube and centrifuged at 12,000 rpm for 2 min, washed with washing buffer, and then eluted using elution buffer and stored at -80 ℃ until subsequent experiments. The quality and integrity of RNA samples were determined using agarose gel electrophoresis. If the 28S and 18S bands were clearly observed in the sample and the intensity of the 28S band was more than 2-fold that of the 18S band, we considered the quality and integrity of RNA samples as acceptable for further experiments. 

### Reverse transcription and quantitative real-time polymerase chain reaction (qPCR)

Reverse transcription and qPCR were performed using the UltraSYBR One-Step RT-qPCR Kit according to the manufacturer’s instructions (CW0659, CWBio, Beijing, China). Briefly, the reaction mixture was prepared as recommended, and the primers used were as follows: *IL-1b*: Forward: 5’-GACTTCACCATGGAACCCGT-3’, reverse: 5’-GGAGACTGCCCATTCTCGAC-3’; *IL-6*: Forward: 5’-ACCCCAACTTCCAATGCTCT-3’, reverse: 5’-GGTTTGCCGAGTAGACCTC-3’; *TNFA*: Forward: 5’-AACACACGAGACGCTGAAGT-3’, reverse: 5’-TCCAGTGAGTTCCGAAAGCC-3’; *PTGS2*: Forward: 5’-CACGGACTTGCTCACTTTGTT-3’, reverse: 5’-AAGCGTTTGCGGTACTCATT-3’. The reaction was performed using the following steps: reverse transcription at 45 ℃ for 10 min, pre-degeneration at 95 ℃ for 5 min. Subsequently, the following steps were repeated for 36 cycles: denaturation at 95 ℃ for 15 s, annealing at 58 ℃ for 30 s and extension at 72 ℃ for 30 s. Each experiment was replicated thrice independently, and GAPDH was used as an internal control. The expression of each gene was determined using the 2^-ΔΔCq^ method (Grimholt et al., 2013). 

### Protein collection and western blot analysis

The cells and brain tissues were cultured and grouped as previously described. After treatment, cells and brain tissues were lysed with lysis buffer (8 M Urea, 50 mM IAA, 10 mM DTT and a proteinase inhibitor cocktail). After centrifugation, the samples were collected into a new tube, and the concentration of proteins was determined using the BCA assay. Protein samples (60 μg) were loaded and separated by 10% SDS-PAGE, and proteins were transferred onto nitrocellulose membranes. Membranes were then incubated with 5% skim milk for 1 h at room temperature, followed by incubation with primary antibodies at 4 ℃ overnight. After incubation with a secondary antibody for 1 h at room temperature, the gray value of proteins was determined using a chemiluminescent immunoassay, and the expression of each target protein was measured using IPP 6.0. 

### Enzyme-linked immunosorbent assay (ELISA)

ELISA was performed according to the ELISA kit protocol. Briefly, serum samples and cultured cell media were added into each well of a 96-well plate, and the samples were incubated with an antibody at room temperature for 1 h. Afterward, samples were washed with washing buffer thrice, incubated with TMB substrate for 10 min, and the OD value at 450 nm was measured using a SpectraMax iD3 microplate reader (Molecular Devices).

### Statistical analysis

Data of these experiments are presented as mean ± SEM. Each experiment was replicated thrice independently. Differences between groups were analyzed using one-way ANOVA in SPSS (version 22.0, SPSS Inc, Chicago, IL, USA). Statistical significance was set at *P* < 0.05. 

## Results

### Detection of cell viability in each group using the CCK-8 assay

**Figure 1 -:**
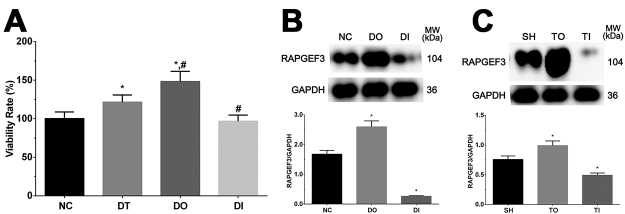
Viability rate of glial cells in NC, DT, DO and DI group. (A) Viability rate of glial cells. (B) Expression of RAPGEF3 in NC, DO and DI group of glial cells without treatment of dezocine. (C) Expression of RAPGEF3 in SH, TO and TI group of rats model without treatment of dezocine. Data was presented as mean ± SD, each experiment was repeated for three times. *P<0.05 vs NC group of cells or SH group of rats. #P<0.05 vs signal dezocine treatment group. NC: normal group, DT: dezocine treatment group, DO: dezocine treatment combined with RAPGEF3 overexpression group, DI: dezocine treatment combined with RAPGEF3 inhibition group. SH: sham group, TD: dezocine treatment group, TO: dezocine treatment combined with RAPGEF3 overexpression group, TI: dezocine treatment combined with RAPGEF3 inhibition group.

As shown in Figure 1A, the viability rate was significantly increased in the DT and DO groups (P<0.05) compared with that in the NC group, significantly increased in the DO group (P<0.05) and significantly decreased in the DI group (P<0.05) compared with that in the DT group. Therefore, the results showed that dezocine increased the viability rate of cells, and overexpression of RAPGEF3 enhanced this effect. 

### Western blotting analysis of RAPGEF3 expression in each group of cell and rat models

As shown in Figures 1B and 1C, the expression of RAPGEF3 was significantly increased in the overexpression group and significantly decreased in the inhibition group (P<0.05), indicating that the RAPGEF3 overexpression and inhibition model was successfully constructed in cells and rats.

### The effect of overexpression of RAPGEF3 combined with dezocine on the cognitive performance of rats in the acquisition trial and MWM probe test

**Figure 2 -:**
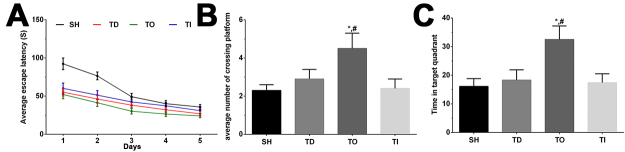
Detection of memory function using MWM. (A) The escape latency of rats in each group in a continuous 5 days. (B) The average number of crossing platform of rats in each group. (C) The time in target quadrant of rats in each group. Data was presented as mean ± SD. Each experiment was repeated for three times independently. *P<0.05 compared with SH group. #P<0.05 compared with TD group. NC: normal group, DT: dezocine treatment group, DO: dezocine treatment combined with RAPGEF3 overexpression group, DI: dezocine treatment combined with RAPGEF3 inhibition group. SH: sham group, TD: dezocine treatment group, TO: dezocine treatment combined with RAPGEF3 overexpression group, TI: dezocine treatment combined with RAPGEF3 inhibition group.

As shown in Figure 2, the performance of rats in each group in the MWM test is presented. The escape latency was significantly reduced in the TO group (P<0.05), and the average number of crossing platforms and the time spent in the target quadrant were significantly higher in the TO group than those in the SH and TD groups. These results indicate that dezocine treatment enhances memory function, and overexpression of RAPGEF3 enhances this trend.

### qPCR analysis of inflammation-related molecule expression for each group in cell and rats models

**Figure 3 -:**
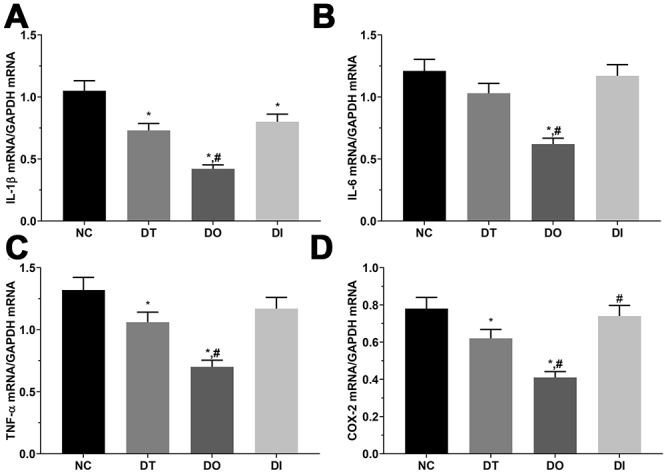
Detection of pro-inflammatory factors in glial cells. (A) Expression of *IL-1b* mRNA in NC, DT, DO and DI group of glial cells. (B) Expression of *IL-6* mRNA in NC, DT, DO and DI group of glial cells. (C) Expression of *TNF-A* in NC, DT, DO and DI group of glial cells. (D) Expression of *PTGS2* in NC, DT, DO and DI group of glial cells. Data was presented as mean ± SD, each experiment was repeated for three times. *P<0.05 vs NC group of cells. #P<0.05 vs signal dezocine treatment group. NC: normal group, DT: dezocine treatment group, DO: dezocine treatment combined with RAPGEF3 overexpression group, DI: dezocine treatment combined with RAPGEF3 inhibition group. SH: sham group, TD: dezocine treatment group, TO: dezocine treatment combined with RAPGEF3 overexpression group, TI: dezocine treatment combined with RAPGEF3 inhibition group.

**Figure 4 -:**
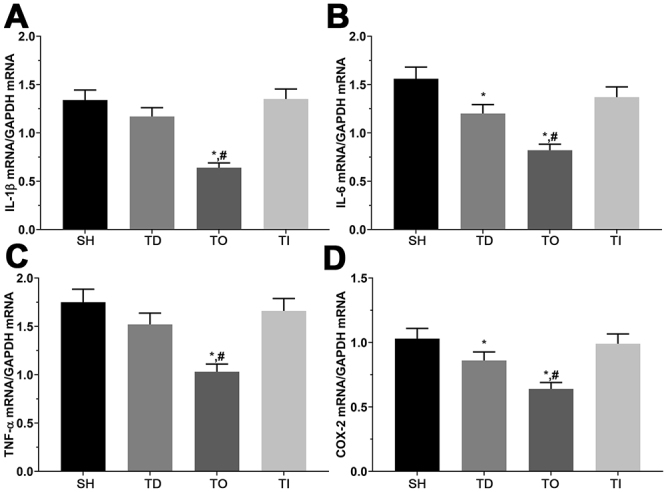
Detection of pro-inflammatory factors in brain tissues of rats. (A) Expression of *IL-1b* mRNA in SH, TD, TO and TI group of rats. (B) Expression of *IL-6* mRNA in SH, TD, TO and TI group of rats. (C) Expression of *TNF-A* mRNA in SH, TD, TO and TI group of rats. (D) Expression of *PTGS2* mRNA in SH, TD, TO and TI group of rats. Data was presented as mean ± SD, each experiment was repeated for three times. *P<0.05 vs SH group of rats. #P<0.05 vs signal dezocine treatment group. NC: normal group, DT: dezocine treatment group, DO: dezocine treatment combined with RAPGEF3 overexpression group, DI: dezocine treatment combined with RAPGEF3 inhibition group. SH: sham group, TD: dezocine treatment group, TO: dezocine treatment combined with RAPGEF3 overexpression group, TI: dezocine treatment combined with RAPGEF3 inhibition group.

As shown in Figures 3 and 4, the expression of *IL-1b*, *IL-6*, *TNFA* and *PTGS2* was determined using qPCR. The fold change of *IL-1b* was 1.00 ± 0.08, 0.70 ± 0.06, 0.40 ± 0.03, and 0.76 ± 0.06 in the NC, DT, DO, and DI groups, respectively, and was significantly decreased in all treatment groups relative to that in the NC group (P<0.05), and in the DO group compared with that in the DT group (P<0.05) in the cell model. The fold change of *IL-1b* was 1.00 ± 0.07, 0.87 ± 0.07, 0.48 ± 0.02, and 1.00 ± 0.07 was significantly decreased in the TO group compared with the SH and TD groups (P<0.05) in the rat model. The fold change of *IL-6* was 1.00 ± 0.07, 0.85 ± 0.06, 0.51 ± 0.04, and 0.97 ± 0.07, and was significantly decreased in the DO group compared with that in the NC and DT groups (P<0.05). The fold change of *IL-6* was 1.00 ± 0.08, 0.77 ± 0.06, 0.53 ± 0.04, and 0.88 ± 0.07 and was significantly decreased in the TD and TO groups compared with that in the SH group (P<0.05) in cell model, as well as in the TO group compared with that in the TD group (P<0.05) in the rat model. The fold change of *TNF-A* was 1.00 ± 0.08, 0.80 ± 0.06, 0.55 ± 0.04 and 0.89 ± 0.07, respectively, and was significantly decreased in the DT and DO groups than that in the NC group (P<0.05), as well as in the DO group compared with that in the DT group (P<0.05) in the cell model. The fold change of *TNF-A* was 1.00 ± 0.07, 0.87 ± 0.07, 0.59 ± 0.05, and 0.95 ± 0.07, respectively, and was significantly decreased in the TO group relative to that in the SH and TD groups (P<0.05) in the rat model. The fold change of *PTGS2* was 1.00 ± 0.08, 0.79 ± 0.06, 0.53 ± 0.04, and 0.95 ± 0.08, and was significantly decreased in the DT and DO groups compared with that in the NC group (P<0.05), significantly decreased in the DO group and significantly increased in the DI group than that in the DT group (P<0.05) in the cell model. The fold change of *PTGS2* was 1.00 ± 0.07, 0.83 ± 0.07, 0.62 ± 0.05, and 0.96 ± 0.08, and was significantly decreased in the DT and DO groups compared with that in the SH groups (P<0.05), as well as in the DO group compared with that in the DT group (P<0.05). 

### Western blotting analysis of inflammation-related proteins in each group of cells and the rat model

**Figure 5 -:**
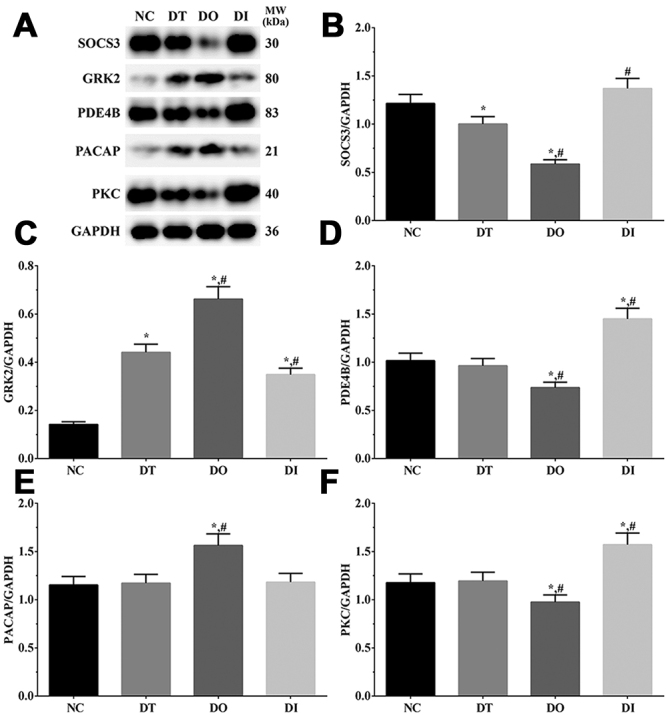
Detection of neuron protective factors in glial cells. (A) Western blotting analysis of SOCS3, GRK2, PDE4B, PCACP and PKC in glial cells. (B-F) Quantitative analysis of each target protein. Data was presented as mean ± SD, each experiment was repeated for three times. *P<0.05 vs NC group of cells. #P<0.05 vs signal dezocine treatment group. NC: normal group, DT: dezocine treatment group, DO: dezocine treatment combined with RAPGEF3 overexpression group, DI: dezocine treatment combined with RAPGEF3 inhibition group. SH: sham group, TD: dezocine treatment group, TO: dezocine treatment combined with RAPGEF3 overexpression group, TI: dezocine treatment combined with RAPGEF3 inhibition group.

**Figure 6 -:**
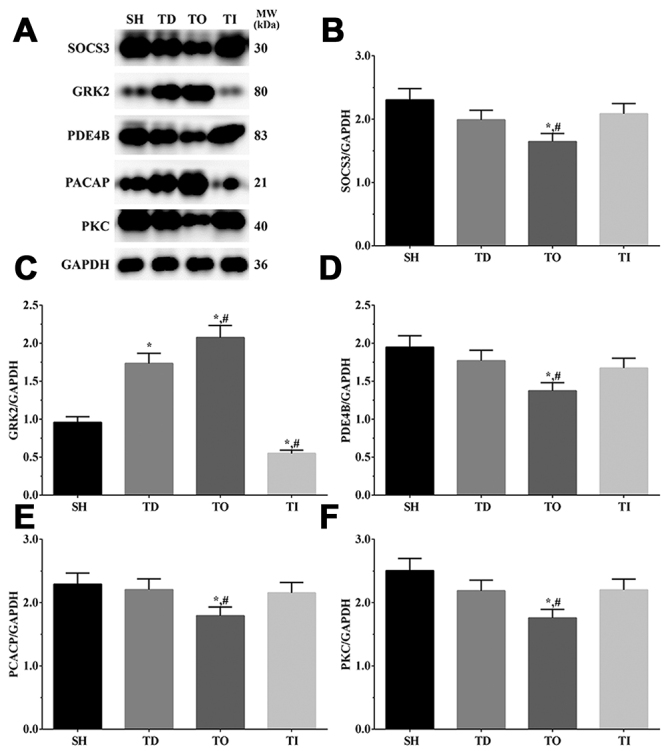
Detection of neuron protective factors in brain tissue of rats. (A) Western blotting analysis of SOCS3, GRK2, PDE4B, PCACP and PKC in glial cells. (B-F) Quantitative analysis of each target protein. Data was presented as mean ± SD, each experiment was repeated for three times. *P<0.05 vs SH group of rats. #P<0.05 vs signal dezocine treatment group. NC: normal group, DT: dezocine treatment group, DO: dezocine treatment combined with RAPGEF3 overexpression group, DI: dezocine treatment combined with RAPGEF3 inhibition group. SH: sham group, TD: dezocine treatment group, TO: dezocine treatment combined with RAPGEF3 overexpression group, TI: dezocine treatment combined with RAPGEF3 inhibition group.

As shown in Figures 5 and 6, the expression of inflammation-related proteins in cell and rat models was determined using western blotting analysis. Briefly, the expression of SOCS3 was significantly decreased in the DT and DO groups than that in the NC group (P<0.05), significantly decreased in the DO group and significantly increased in the DI group relative to that in the DT group (P<0.05) in the cell model, whereas it was significantly decreased in the DO group compared with that in the SH and DT groups (P<0.05) in the rat model. The expression of GRK2 was significantly increased in all treatment groups than that in the NC group (P<0.05) in the cell model, significantly increased in the DO group, and significantly decreased in the DI group compared with that in the DT group (P<0.05) in the cell model. Additionally, GRK2 expressoin was significantly increased in the TD and TO groups and significantly decreased in the TI group relative to that in the SH group (P<0.05), significantly increased in the TO group, and significantly decreased in the TI group compared with that in the TD group (P<0.05) in the rat model. The expression of PDE4B was significantly decreased in the DO group and significantly increased in the DI group compared with that in the NC and DT groups (P<0.05) in the cell model and was significantly decreased in the TO group compared with that in the SH and TD groups (P<0.05) in the rat model. The expression of PACAP was significantly higher in the DO group than that in the NC and DT groups (P<0.05) in the cell model and was significantly higher in the TO group than that in the SH and TD groups (P<0.05) in the rat model. The expression of PKC was significantly decreased in the DO group and significantly increased in the DI group compared with that in the NC and DT groups (P<0.05) in the cell model, and was significantly lower in the TO group than that in the SH and TD groups (P<0.05) in the rat model. 

### Determination of Ras/MAPK signaling pathway activation in cell and rat models using western blotting analysis

**Figure 7 -:**
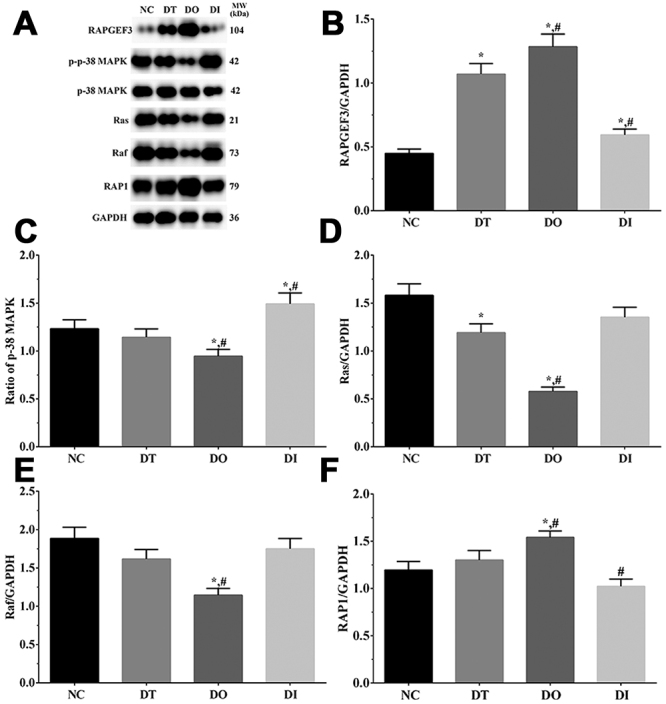
Activation of Ras/Raf/MAPK in glial cells. (A) Western blotting analysis of RAPGEF3, p-38 MAPK, Ras, Raf and PKC in glial cells. (B-F) Quantitative analysis of each target protein. Data was presented as mean ± SD, each experiment was repeated for three times. *P<0.05 vs NC group of cells. #P<0.05 vs signal dezocine treatment group. NC: normal group, DT: dezocine treatment group, DO: dezocine treatment combined with RAPGEF3 overexpression group, DI: dezocine treatment combined with RAPGEF3 inhibition group. SH: sham group, TD: dezocine treatment group, TO: dezocine treatment combined with RAPGEF3 overexpression group, TI: dezocine treatment combined with RAPGEF3 inhibition group.

**Figure 8 -:**
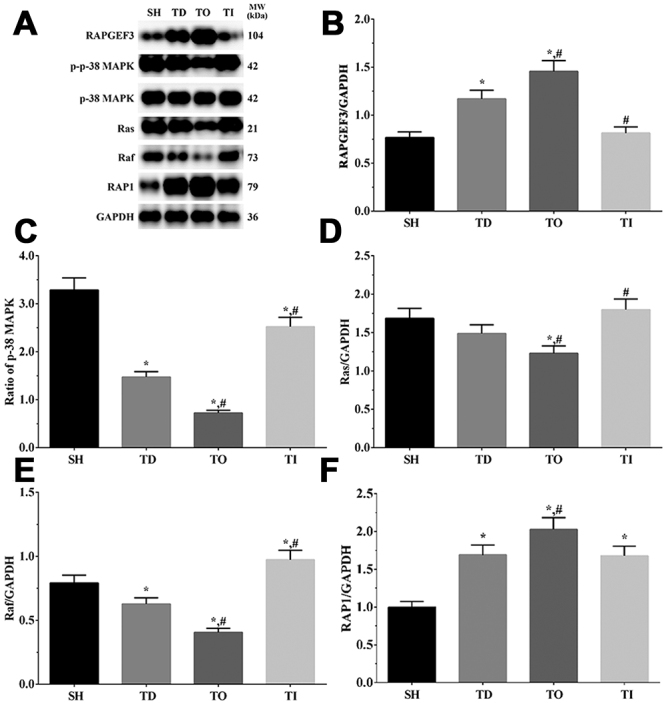
Activation of Ras/Raf/MAPK in brain tissue of rats. (A) Western blotting analysis of RAPGEF3, p-38 MAPK, Ras, Raf and PKC in glial cells. (B-F) Quantitative analysis of each target protein. Data was presented as mean ± SD, each experiment was repeated for three times. *P<0.05 vs SH group of rats. #P<0.05 vs signal dezocine treatment group. NC: normal group, DT: dezocine treatment group, DO: dezocine treatment combined with RAPGEF3 overexpression group, DI: dezocine treatment combined with RAPGEF3 inhibition group. SH: sham group, TD: dezocine treatment group, TO: dezocine treatment combined with RAPGEF3 overexpression group, TI: dezocine treatment combined with RAPGEF3 inhibition group.

As shown in Figures 7 and 8, the activation of the Ras/MAPK signaling pathway in cell and rat models was determined using western blot analysis. The expression of RAPGEF3 was significantly increased in all treatment groups compared with that in the NC group (P<0.05), significantly increased in the DO group, and significantly decreased in the DI group relative to that in the DT group (P<0.05) in the cell model. Furthermore, RAPGEF3 expression was significantly increased in the TD and TO groups compared with that in the SH group (P<0.05), significantly increased in the TO group and significantly decreased in the TI group compared with that in the TD group (P<0.05) in the rat model. The expression of Ras was significantly decreased in the DT and DO groups compared with that in the NC group (P<0.05) and significantly decreased in the DO group compared with that in the DT group (P<0.05) in the cell model. Additionally, Ras expression was significantly decreased in the TO group than that in the SH and TD groups (P<0.05) and significantly increased in the TI group than that in the TD group (P<0.05) in the rat model. The expression of Raf was significantly decreased in the DO group compared with that in the NC and DT groups (P<0.05) in the cell model, as well as in the TD and TO groups and significantly increased in the TI group compared with that in the SH group (P<0.05). Furthermore, Raf expression was significantly decreased in the TO group and significantly increased in the TI group compared with that in the TD group (P<0.05) in the rat model. The expression of RAP1 was significantly increased in the DO group compared with that in the NC and DT groups and significantly decreased in the DI group compared with that in the DT group (P<0.05) in the cell model. Furthermore, RAP1 expression was significantly increased in all treatment groups than that in the SH group and significantly increased in the TO group than that in the TD group (P<0.05) in the rat model. The ratio of p-p38 MAPK/p-38 MAPK was significantly decreased in the DO group and significantly increased in the DI group compared with that in the NC and DT groups (P<0.05) in the cell model. Additionally, the ratio of p-p38 MAPK/p-38 MAPK was significantly decreased in all treatment groups compared with that in the SH group (P<0.05), significantly decreased in the TO group, and significantly increased in the TI group relative to that in the TD group (P<0.05) in the rat model. 

### Determination of the concentration of inflammatory cytokines in cultured cell media and serum samples of rats

**Figure 9 -:**
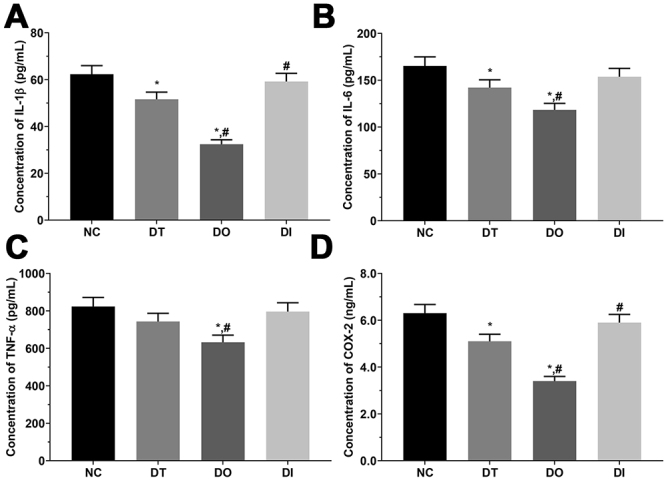
Concentration of pro-inflammatory factor in cultured medium of glial cells. (A) Concentration of IL-1β mRNA in NC, DT, DO and DI group of glial cells. (B) Concentration of IL-6 mRNA in NC, DT, DO and DI group of glial cells. (C) Concentration of TNF-α in NC, DT, DO and DI group of glial cells. (D) Concentration of COX-2 in NC, DT, DO and DI group of glial cells. Data was presented as mean ± SD, each experiment was repeated for three times. *P<0.05 vs NC group of cells. #P<0.05 vs signal dezocine treatment group. NC: normal group, DT: dezocine treatment group, DO: dezocine treatment combined with RAPGEF3 overexpression group, DI: dezocine treatment combined with RAPGEF3 inhibition group. SH: sham group, TD: dezocine treatment group, TO: dezocine treatment combined with RAPGEF3 overexpression group, TI: dezocine treatment combined with RAPGEF3 inhibition group.

**Figure 10 -:**
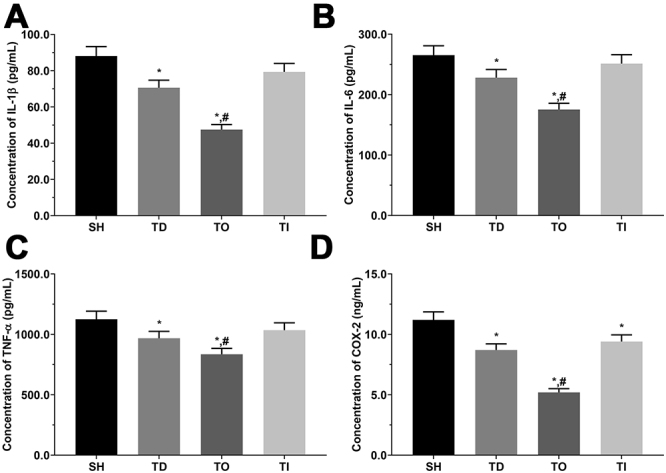
Concentration of pro-inflammatory factor in serum samples of rats. (A) Concentration of IL-1β mRNA in NC, DT, DO and DI group of rats. (B) Concentration of IL-6 mRNA in NC, DT, DO and DI group of rats. (C) Concentration of TNF-α in NC, DT, DO and DI group of rats. (D) Concentration of COX-2 in NC, DT, DO and DI group of rats. Data was presented as mean ± SD, each experiment was repeated for three times. *P<0.05 vs SH group of rats. #P<0.05 vs signal dezocine treatment group. NC: normal group, DT: dezocine treatment group, DO: dezocine treatment combined with RAPGEF3 overexpression group, DI: dezocine treatment combined with RAPGEF3 inhibition group. SH: sham group, TD: dezocine treatment group, TO: dezocine treatment combined with RAPGEF3 overexpression group, TI: dezocine treatment combined with RAPGEF3 inhibition group.

As shown in Figures 9 and 10, the concentration of IL-1β was significantly decreased in the DT and DO groups compared with that in the NC group (P<0.05), significantly decreased in the DO group, and significantly increased in the DI group compared with that in the DT group (P<0.05) in the cell model. In addition, IL-1β concentration was significantly decreased in the TD and TO groups than that in the SH group and significantly decreased in the TO group than that in the TD group (P<0.05) in the rat model. The concentration of IL-6 was significantly lower in the DT and DO groups than that in the NC group, and significantly decreased in the DO group compared with that in the DT group (P<0.05) in the cell model, and presented a similar trend in the rat model. The concentration of TNF-α was significantly lower in the DO group than that in the NC and DT groups (P<0.05) in the cell model. Furthermore, TNF-α concentration was significantly decreased in the TD and TO groups compared with that in the SH group (P<0.05), and significantly decreased in the TO group compared with that in the TD group (P<0.05) in the rat model. The concentration of COX-2 was significantly decreased in the DT and DO groups compared with that in the NC group (P<0.05), significantly decreased in the DO group and significantly increased in the DI group compared with that in the DT group (P<0.05) in the cell model. Furthermore, COX-2 concentration was significantly decreased in all treatment groups than that in the SH group and significantly decreased in the TO group relative to that in the TD group (P<0.05) in the rat model. 

## Discussion

Injury of the peripheral nervous system induced by traumatic, metabolic, and toxic factors, is the leading cause of neuropathic pain development (Ji et al., 2014). Dezocine has been widely used in clinical trials, and has been found to relieve neuropathic pain with high safety (Ding and White, 1992). A previous study demonstrated that in a CCI mouse model, pain sensitivity could be attenuated by dezocine. This effect presented no signs of tolerance, and the authors surmised that dezocine could be considered an alternative medication for treating NP (Wu et al., 2019). Moreover, a recent study reported that subcutaneous injection of dezocine inhibited NP in patients with cancer in a time-dependent manner (Mao et al., 2020). However, the effect of dezocine in treating NP is not fully clear. Rap guanine nucleotide exchange factor 3 (RAPGEF3) is a regulator of the Ras family, especially Rap1 and Rap2, and is involved in cellular survival, cytokine secretion, and cell migration (Kumar et al., 2017). Based on these characteristics, we hypothesized that RAPGEF3 might contribute to the therapeutic effect of dezocine. 

During chronic pain, inflammatory mediators are continuously produced, and pro-inflammatory factors, including TNF-α and IL-1β, induce pain through activation and sensitization of nociceptors (Ji et al., 2018). The cyclooxygenase (COX) pathway participates in the precursor substrate of arachidonic acid (AA) and produces prostaglandins (PGs) that play an important role in regulating the inflammatory response. Production of IL-1β induces COX-2 expression, resulting in the production of prostaglandin (PG)E2 (Cheng et al., 2013); furthermore, IL-6 and TNF-α expression is activated, inducing the activation of the inflammatory response process. IL-6 plays a vital role in regulating immune diseases, infections, and metabolic diseases (Rose-John S., 2018. TNF-α also regulates the inflammatory response process and cytokine activation network (Balkwill, 2009). The expression of COX-2 is also regulated by inflammatory stimuli, such as IL-1, IL-6, and TNF-α, further inducing the expression of downstream molecules involved in inflammation and the neoplastic growth process (Song et al., 1998). Here, using qPCR and ELISA, we found that the expression of these pro-inflammatory factors such as IL-1β, IL-6, TNF-α, and COX-2, as well as their concentration in serum and culture medium, were decreased, indicating that dezocine treatment decreased the expression and secretion of these cytokines, and overexpression of RAPGEF3 further enhanced this effect, leading to the inhibition of inflammatory response process in NP models, indicating a potential therapeutic effect. 

Suppressors of cytokine signaling (SOCS) are known regulators of the Janus kinase (JAK)/ signal transducer and activators of the transcription (STAT) pathway through a negative feedback mechanism (Polizzotto et al., 2000). After damage to neuronal cells, SOCS3 expression is normally upregulated, limiting the function of the ciliary neurotrophic factor (CNTF) on neuron cells, as injection of CNTF in SOCS3 deletion mice induced robust axon regeneration (Smith et al., 2009) and overexpression of SOCS3 diminished the axon regeneration induced by CNTF (Hellström *et al.*, 2011). A previous study found that RAPGEF3 could inhibit the inflammation process induced by IL-6 through upregulation of SOCS3 (Sands et al., 2006). Another study reported that inhibition of RAPGEF3 leads to the downregulation of PDE4, which in turn inhibits the vascular inflammation process (Michael et al., 2015). Using RAPGEF3 knockdown mice, a recent study showed that RAPGEF3 plays a critical role in regulating chronic inflammatory pain via the regulation of RAPGEF3 phosphorylation through GRK2 (Pooja et al., 2016). Furthermore, it was observed that the interaction between GRK2 and RAPGEF3 contributes to chronic pain in RAPGEF3 knockout mice and that increased GRK2 expression inhibitschronic pain in a chronic inflammatory pain model (Wang et al., 2013). G protein-coupled receptor kinase 2 (GRK2) is a member of the G protein coupled receptor (GPCR) kinase family that regulates the function of inflammatory cells and the secretion of inflammatory cytokines under normal conditions (Tang et al., 2017). Under the activation of inﬂammatory immune response conditions, expression, and activation of GRK2 are disrupted, resulting in the production of inflammatory cytokines and cell damage (Taguchi et al., 2017). Phosphodiesterase 4 B (PDE4B) is an enzyme that regulates the intercellular concentration of cAMP (Li et al., 2018). A previous study determined that inhibition of PDE4B expression leads to the accumulation of cAMP, further activation of RAPGEF3 and protection against neuronal apoptosis and inflammatory responses in neuronal cells (Zhang *et al.*, 2019). PACAP was first discovered in 1989 in an ovine hypothalamic extract and has been reported to participate in various developmental processes (Basille et al., 2000). After injury to the nervous system, the expression of several growth factors was elevated, as well as PCACP expression (Johanson et al., 2011), promoting nervous system regeneration. Thus, PCACP is regarded as a neuroprotective factor. It has previously been established that using PKCα antagonists or gene deletion alleviated nociceptive hypersensitivity (Park et al., 2009). Protein kinases, such as MAPK and PKC, are critical nociceptors regulators, resulting in peripheral sensitization induction and maintenance (Ji et al., 2009). Further studies have shown that upregulation of PKCα is mediated by direct or indirect activation of the mitogen-activated protein kinase (MAPK) pathway (Yang et al., 2007). Here, we found that the expression of GRK2 and PACAP was increased after overexpression of RAPGEF3, while the expression of SOCS3, PDE4B and PKC was decreased, indicating that overexpression of RAPGEF3 promotes the protective effect of dezocine. 

RAPGEF3 is a sensor of the cellular second messenger cAMP and acts as the exchange factor of the Rap and Ras families; increased cAMP expression promotes the translocation of RAPGEF3 to the cellular membrane, further inducing Rap1 activation (Ponsioen et al., 2009). Rap1 is a small molecular weight GTPase that belongs to the subfamily of the Ras family and has been shown to regulate several biological processes, including cellular growth, adhesion, and apoptosis (Takai et al., 2001). Activation of RAP1 induced by RAPGEF3 further leads to the activation of MAPK, resulting in the opening of K^+^ channels to promote cellular proliferation (Han et al., 2006). In addition, a previous study reported that the EPAC1/RAP1 signaling pathway promotes cancer cell proliferation by contributing to the glucose uptake and metabolism ability of cells (Onodera et al., 2014). Here, we observed that RAPGEF3 overexpression leads to an increase in the cellular viability rate, which might be induced by RAP1 and its downstream molecules. The Ras/Raf/MAPK signaling pathway is the central regulator of multiple biochemical signals and regulates numerous cellular processes, including proliferation, differentiation and development (Kawasaki et al., 1998). Accumulating evidence identifies the association between Ras/Raf/MAPK signaling pathways and recovery of neuropsychiatric disorders (Zhong, 2016). A recent study indicated that inhibition of the Ras/Raf/MAPK signaling pathway significantly enhances the migration of neurons in mouse models (Xu et al., 2016b), and also resulted in a greater restoration of dendritic spine density (Kasai et al., 2010), which is essential for synaptic function and recovery of neuronal cells from injury. Furthermore, a previous study indicated that activation of the Ras/MAPK signaling pathway impairs the formation of synapses, and inhibits the development of mature spines (Cao et al., 2013). 

## Conclusion

Here, we demonstrated that dezocine treatment combined with overexpression of RAPGEF3 significantly increased the viability of cells, decreased the expression of inflammation-related cytokines at the transcriptional level and the concentration of inflammatory cytokines in cultured medium and serum samples of cells and rats. In contrast, the expression of anti-inflammatory and neuroprotective proteins increased. We further revealed that these effects might be mediated by inhibition of the Ras/Raf/MAPK signaling pathway. Thus, we hypothesized that dezocine treatment combined with RAPGEF3 overexpression might have a therapeutic effect on NP by increasing the proliferation ability of neuronal cells. However, the present results were limited by the small sample size of the rats and the limitation of the research methods. Thus, further experimentation with human samples is required. 
